# High-cost health care users in Ontario, Canada: demographic, socio-economic, and health status characteristics

**DOI:** 10.1186/s12913-014-0532-2

**Published:** 2014-10-31

**Authors:** Laura C Rosella, Tiffany Fitzpatrick, Walter P Wodchis, Andrew Calzavara, Heather Manson, Vivek Goel

**Affiliations:** Dalla Lana School of Public Health, University of Toronto, 155 College Street, Suite 600, Toronto, ON M5T 3M7 Canada; Institute for Clinical Evaluative Sciences, Room 424, 155 College Street, Toronto, ON M5T 3M6, Canada; Public Health Ontario, 480 University Avenue, Suite 300, Toronto, ON M5G 1V2 Canada; Institute of Health Management Policy and Evaluation, University of Toronto, Suite 425, 155 College Street, Toronto, ON M5T 3M7 Canada; Toronto Rehabilitation Institute, 550 University Avenue, Toronto, ON M5G 2A2 Canada

**Keywords:** Health care utilization, High-cost users, Upstream determinants, Population perspective, Canadian Community Health Survey

## Abstract

**Background:**

Health care spending is overwhelmingly concentrated within a very small proportion of the population, referred to as the high-cost users (HCU). To date, research on HCU has been limited in scope, focusing mostly on those characteristics available through administrative databases, which have been largely clinical in nature, or have relied on ecological measures of socio-demographics. This study links population health surveys to administrative data, allowing for the investigation of a broad range of individual-level characteristics and provides a more thorough characterization of community-dwelling HCU across demographic, social, behavioral and clinical characteristics.

**Methods:**

We linked three cycles of the Canadian Community Health Survey (CCHS) to medical claim data for the years 2003–2008 for Ontario, Canada. Participants were ranked according to gradients of cost (Top 1%, Top 2-5%, Top 6-50% and Bottom 50%) and multinomial logistic regression was used to investigate a wide range of factors, including health behaviors and socio-demographics, likely associated with HCU status.

**Results:**

Using a total sample of 91,223 adults (18 and older), we found that HCU status was strongly associated with being older, having multiple chronic conditions, and reporting poorer self-perceived health. Specifically, in the fully-adjusted model, poor self-rated health (vs. good) was associated with a 26-fold increase in odds of becoming a Top 1% HCU (vs. Bottom 50% user) [95% CI: (18.9, 36.9)]. Further, HCU tended to be of lower socio-economic status, former daily smokers, physically inactive, current non-drinkers, and obese.

**Conclusions:**

The results of this study have provided valuable insights into the broader characteristics of community-dwelling HCU, including unique demographic and behavioral characteristics. Additionally, strong associations with self-reported clinical variables, such as self-rated general and mental health, highlight the importance of the patient perspective for HCU. These findings have the potential to inform policies for health care and public health, particularly in light of increasing decision-maker attention in the sustainability of the health care system, improving patient outcomes and, more generally, in order to achieve the common goal of improving population health outcomes.

## Background

It is well known that health care spending is overwhelmingly concentrated; a very small proportion of the population consumes the majority of costs. In 2007/08, the top 1% of health care users in Ontario accounted for one-third of health care spending; the lower 50% of users, on the other hand, consumed a mere 1% of total expenditures [1]. This is not a phenomenon specific to Ontario, nor is it one isolated within Canada’s universal health care system. Indeed, this skewness in health care spending has been documented in nearly every health care system [2-16].

There has been a renewed interest in the so-called “high-cost users” (HCU) of health care in recent years. Policy makers are becoming increasingly concerned with the sustainability of the health care system, quality of care and patient outcomes [13,17-19]. Attention has been placed on programs to manage high-risk groups, such as the elderly and those with multiple comorbidities [18,19]. Research has overwhelmingly focused on improving patient outcomes through better case management and strategies to reduce health care spending. Few, however, have highlighted the importance of identifying the broader determinants of HCU, which can be very influential upon health status. Understanding the HCU population from a broader perspective is paramount to better managing patients who are currently HCU or those on the path to becoming HCU.

To date, the narrow characterization of HCU has been largely due to the limited data on social and behavioral factors linked with utilization outcomes. To overcome this gap, we have linked medical care use data for participants from several cycles of a national population-based survey. This has allowed us to investigate a multitude of individual-level characteristics not available in previous studies. By investigating a broad range of socio-economic, behavioral, and health status characteristics, we aim to more fully characterize HCU living in the community.

## Methods

### Data sources

We linked population-based health administrative data from Ontario, Canada, to participants from multiple cycles of the Canadian Community Health Survey (CCHS) conducted in 2003, 2005, and 2007/8. The CCHS, a cross-sectional survey administered by Statistics Canada, collects self-reported health-related data. Briefly, the CCHS uses a multi-stage sampling survey design to target Canadians 12 years of age or older living in private dwellings. Detailed survey methodology is documented elsewhere [20]. All Ontario residents are covered by a single payer insurance system referred to as the Ontario Health Insurance Plan (OHIP) and all related health care utilization is tracked in the health administrative data.

Health care spending was calculated using administrative data across health care sectors, including in-patient hospital stay, emergency department visits, same day surgery, stays in complex continuing care hospitals and inpatient rehabilitation, inpatient psychiatric admissions, physician payments for patient visits and community laboratory, and prescriptions filled for individuals eligible for the Ontario Drug Benefit (ODB).” A person-centered costing methodology, developed at the Institute for Clinical Evaluative Sciences (ICES), was used to determine total health care spending [21].

Participants were excluded if they could not be successfully linked to administrative data, e.g. not found in the Registered Persons Database (RPDB), a population-based registry maintained by Ontario’s Ministry of Health and Long-term Care; already appeared in a previous cycle of CCHS; or were OHIP-ineligible for the entire observation window. Respondents aged 12 – 18 were also excluded.

Prior health care utilization was captured using a 2-year look-back period from interview date and Aggregated Diagnosis Groups (ADGs) scores were calculated [22]. Briefly, the Johns Hopkins ADGs provide a numeric method for grouping diagnostic codes similar to terms of severity and likelihood of persistence. Their use has previously been validated in the adult Ontario population [22]. All other baseline characteristics were captured from interview variables including household income, demographics, health behaviors and medical history (e.g. health status, chronic conditions, having a regular medical doctor). For the outcome, we calculated costs based on utilization for the year following interview date and ranked individuals according to gradients of cost within each CCHS cohort (1, 2–5, 6–50 and lower 50th percentiles); HCU were defined as the Top 5% of users.

### Statistical analysis

Descriptive analyses (frequency, mean, median) were calculated for all covariates across four utilization ranking groups and the overall cohort population. Multinomial logistic regression models were used to quantify the association between socio-economic, demographic, health and behavioral characteristics with gradients of use, including unadjusted, age-adjusted and fully-adjusted models. Using the Bottom 50% as the referent group, the multinomial models estimated the odds for each of the Top 1%, Top 2-5% and Top 6-50% cost-rank groups. A nominal multinomial model was chosen given the hypothesized differential association between the characteristics of interest and gradients of use. The proportional odds assumption was tested to assess the appropriateness of treating the outcome as categorical, opposed to ordinal; where p <0.05 signifies a violation of the proportional odds assumption (i.e. the response should not be treated as ordered) [23]. All covariates were treated as categorical. Sensitivity analyses were conducted to assess the effect of including additional individual- and ecological-level SES measures. The more parsimonious model was kept if there were non-significant changes to model estimates.

Bootstrap sampling weights, as provided by Statistics Canada, were applied using Balanced Repeated Replication (BRR) to all analyses to adjust for the complex survey design of the CCHS and to produce estimates reflecting a sample of the Ontario population weighted over three time points. Combining cycles of the CCHS in this manner, referred to as the “pooled approach”, allows for an increased sample size, and thus, statistical power [24]. Weighted 95% confidence limits were calculated for all descriptive and regression estimates.

All statistical analyses were performed using SAS version 9.3 (SAS Institute Inc; Cary, NC). Proc SurveyFreq and Proc SurveyMeans were used to conduct descriptive analyses; Proc SurveyLogistic was used for multinomial logistic regression.

### Ethics approval

The study design received ethics approval from the Ethics Review Boards of Public Health Ontario and Sunnybrook Health Sciences Center.

## Results

A total of 101,719 individuals responded to the surveys and were successfully linked to administrative data via provincial insurance health card number; resulting in a linkage rate of approximately 84% among those respondents who agreed to share their data. After applying our exclusion criteria, a sample of 91,223 adult respondents was included in our analysis; with approximately one-third of respondents coming from each CCHS cycle. Exclusions were not significantly different across cycles.

Table 1 provides the weighted distribution of all demographic, socio-economic, health and behavioral characteristics for each of the four utilization groups (Top 1%, Top 2-5%, Top 6-50%, and Bottom 50%) and the overall CCHS weighted Ontario population. Compared to those with lower utilization, community-dwelling HCU (Top 5%) tended to be older, white ethnicity, and have lower household income. HCU more frequently reported having a regular medical doctor, having any chronic disease and had mean ADG scores magnitudes greater than the moderate and low utilization groups. HCU were also more likely to report poorer self-perceived health (both general and mental health) and to have reported consulting a mental health professional in the past year. Furthermore, HCU tended to be overweight or obese, former smokers, physically inactive, and current non-drinkers. All of these associations were more pronounced for the extreme HCU (Top 1%).

**Table 1:** The weighted* distribution of demographic, socio-economic, health and behavioral characteristics across health care expenditure categories using the 2003–2008 CCHS cohorts for adult Ontarians

**Characteristic**	**Overall % (95% CI)**	**Top 1% % (95% CI)**	**Top 2 – 5% % (95% CI)**	**Top 6 – 50% % (95% CI)**	**Bottom 50% % (95% CI)**
Total Population	N =28,529,265	N =285,255	N =1,141,128	N =12,839,271	N =14,263,611
**Socio-economics**					
**Sex (Male)**	48.9 (48.8, 49.0)	50.4 (45.5, 55.4)	43.4 (41.3, 45.6)	39.6 (39.0, 40.1)	57.7 (57.2, 58.3)
**Age group**					
18 – 34	29.3 (29.0, 29.6)	3.6 (1.8, 5.4)	9.8 (8.5, 11.1)	20.6 (20.1, 21.2)	39.1 (38.6, 39.8)
35 – 49	31.8 (31.3, 32.1)	10.9 (7.5, 14.3)	15.2 (13.4, 17.1)	27.1 (26.4, 27.8)	37.7 (36.9, 38.4)
50 – 64	23.0 (22.6, 23.3)	22.5 (18.0, 27.0)	22.9 (21.1, 24.7)	26.3 (25.6, 27.0)	20.0 (19.4, 20.5)
65 – 74	9.3 (9.1, 9.5)	24.1 (20.7, 27.5)	22.4 (20.7, 24.1)	15.5 (15.1, 15.9)	2.3 (2.1, 2.5)
75+	6.7 (6.5, 6.9)	38.8 (34.0, 43.6)	29.7 (27.8, 31.5)	10.4 (10.1, 10.8)	0.84 (0.73, 0.95)
**Ethnicity**					
White	78.0 (77.4, 78.5)	87.1 (82.2, 91.5)	87.2 (85.3, 89.1)	78.5 (77.7, 79.3)	76.6 (75.8, 77.4)
Visible Minority	20.2 (19.6, 20.7)	10.8 (6.5, 15.2)	10.7 (8.9, 12.6)	19.6 (18.8, 20.4)	21.6 (20.8, 22.4)
**Immigrant Status**					
Canadian-Born	67.7 (67.2, 68.3)	68.1 (63.1, 73.0)	68.7 (66.6, 70.8)	64.8 (64.0, 65.7)	70.3 (69.4, 71.1)
Immigrant	32.0 (31.4, 32.5)	31.4 (26.4, 36.3)	30.8 (28.7, 32.9)	34.8 (34.0, 35.7)	29.5 (28.6, 30.3)
**Marital Status**					
Married or Common-Law	64.8 (64.3, 65.2)	58.3 (53.6, 62.9)	61.4 (59.3, 63.6)	68.1 (67.4, 68.8)	62.2 (61.5, 62.9)
Other†	35.2 (34.7, 35.7)	41.7 (37.1, 46.3)	38.6 (36.4, 40.7)	31.9 (31.2, 32.6)	37.8 (37.1, 38.5)
**Household Income (Equivalized)**					
Low	16.4 (16.0, 16.8)	32.6 (28.0, 37.2)	28.1 (26.2, 30.0)	19.2 (18.5, 19.8)	12.7 (12.2, 13.1)
Low-middle	17.8 (17.4, 18.2)	19.4 (15.9, 22.8)	19.4 (17.6, 21.1)	18.5 (17.9, 19.1)	17.0 (16.4, 17.6)
Middle	17.1 (16.7, 17.5)	14.8 (11.6, 17.9)	12.6 (11.2, 13.9)	16.0 (15.5, 16.6)	18.4 (17.9, 19.0)
Middle-High	17.9 (17.5, 18.3)	9.5 ( 7.0, 12.0)	12.2 (10.8, 13.7)	16.6 (16.0, 17.2)	19.7 (19.1, 20.3)
High	18.3 (17.8, 18.8)	8.0 (5.1, 10.9)	11.1 (9.7, 12.6)	16.5 (15.9, 17.1)	20.7 (20.0, 21.3)
**Health Status**					
**Has a regular doctor (Yes)**	91.1(90.8,91.4)	94.9 (92.4, 97.3)	95.7 (94.9, 96.5)	94.7 (94.3, 95.1)	87.5 (87.0, 88.0)
**Has a self-reported chronic disease** ^**T**^	53.4 (52.9, 53.9)	92.3 (90.0, 94.6)	85.3 (83.6, 87.0)	67.3 (66.5, 68.1)	37.5 (36.9, 38.2)
Mean ADG Score**	4.9 (4.9, 5.0)	22.7 (21.2, 24.2)	16.7 (16.1, 17.3)	7.2 (7.0, 7.4)	1.6 (1.5, 1.7)
**Body Mass Index (kg/m** ^**2**^ **)**					
Underweight, BMI <18.5	2.7 (2.5, 2.9)	4.0 (2.6, 5.4)	3.4 (2.6, 4.3)	2.5 (2.3, 2.8)	2.7 (2.4, 3.0)
Normal weight, 18.5 – 24.9	44.6 (44.1, 45.2)	36.0 (31.6, 40.5)	35.7 (33.6, 37.7)	41.8 (41.0, 42.6)	48.0 (47.2, 48.8)
Overweight, 25 – 29.9	33.1 (32.6, 33.5)	31.7 (27.4, 36.1)	32.5 (30.5, 34.5)	32.9 (32.2, 33.6)	33.3 (32.5, 34.0)
Obese, BMI >30	15.6 (15.3, 16.0)	19.8 (16.1, 23.6)	20.9 (19.4, 22.5)	17.7 (17.1, 18.3)	13.2 (12.8, 13.7)
**Self-perceived general health**					
Good (excellent/very/good)	87.6 (87.3, 88.0)	38.8 (34.7, 42.8)	57.1 (55.0, 59.2)	82.9 (82.3, 83.6)	95.3 (95.0, 95.6)
Fair	9.0 (8.7, 9.3)	27.7 (24.1, 31.3)	23.8 (22.0, 25.6)	12.5 (12.0, 13.0)	4.2 (3.9, 4.5)
Poor	3.3 (3.1, 3.6)	33.3 (28.8, 37.8)	19.0 (17.1, 20.9)	4.5 (4.1, 4.9)	0.47 (0.39, 0.55)
**Self-perceived mental health**					
Good (excellent/very/good)	92.3 (92.0, 92.7)	70.6 (66.2, 75.0)	80.2 (78.5, 81.8)	90.6 (90.1, 91.1)	95.3 (94.9, 95.7)
Not Good (fair/poor)	5.2 (5.0, 5.5)	14.5 (10.9, 18.2)	11.2 (9.8, 12.5)	6.8 (6.4, 7.3)	3.2 (2.9, 3.4)
**Consulted a mental health professional (past 12 months)**					
Yes	8.9 (8.6, 9.2)	14.4 (10.9, 17.9)	13.3 (11.9, 14.7)	11.7 (11.2, 12.1)	5.9 (5.5, 6.3)
No	88.6 (88.2, 88.9)	70.9 (66.5, 75.3)	78.0 (76.2, 79.8)	85.6 (85.0, 86.1)	92.5 (92.0, 92.9)
**Behavioral**					
**Life Stress**					
High (Quite a bit or extreme)	23.3 (22.8, 23.7)	25.8 (21.3, 30.3)	24.3 (22.4, 26.1)	23.5 (22.9, 24.2)	22.9 (22.2, 23.6)
Low (A bit, not very, none)	76.4 (76.0, 76.9)	73.2 (68.6, 77.8)	74.8 (72.9, 76.7)	76.1 (75.5, 76.8)	76.9 (76.2, 77.6)
**Smoking status**					
Heavy smoker (1+ packs/day)	6.5 (6.3, 6.8)	9.1 (6.2, 11.9)	8.0 (6.9, 9.1)	5.6 (5.3, 6.0)	7.1 (6.8, 7.5)
Light smoker (<1 pack/day)	16.1 (15.7, 16.5)	9.3 (7.1, 11.4)	11.3 (10.0, 12.5)	14.0 (13.5, 14.6)	18.5 (17.9, 19.2)
Former (Daily) smoker	23.5 (23.1, 24.0)	39.2 (34.5, 44.0)	36.1 (34.1, 38.1)	27.3 (26.6, 27.9)	18.9 (18.3, 19.4)
Non-smoker	53.7 (52.2, 54.3)	42.1 (37.3, 46.9)	44.5 (42.4, 46.6)	52.9 (52.1, 53.7)	55.4 (54.6, 56.2)
**Physical activity**					
Inactive (<1.5 METs/day)	49.5 (48.8, 50.1)	59.4 (55.0, 63.9)	55.8 (53.6, 58.0)	52.0 (51.1, 52.8)	46.5 (45.6, 47.3)
Active (1.5+ METs/day)	48.2 (47.5, 48.8)	25.9 (21.9, 29.8)	35.7 (33.5, 37.9)	45.5 (44.6, 46.3)	52.0 (51.1, 52.9)
**Alcohol consumption (past year)†**					
Current Drinker (any)	79.6 (79.1, 80.1)	57.4 (52.4, 62.4)	64.6 (62.4, 66.7)	76.2 (75.5, 77.0)	84.4 (83.7, 85.0)
Current Non-Drinker	20.3 (19.7, 20.8)	42.5 (37.5, 47.6)	35.3 (33.2, 37.5)	23.6 (22.9, 24.4)	15.6 (14.9, 16.2)

*Weighted using bootstrap weights provided by Statistics Canada. †Other: Divorced, separated, widowed or single. ^T^Chronic conditions common to all three CCHS cycles: asthma, arthritis, back problems, migraines, COPD or emphysema, diabetes, high blood pressure, heart disease, cancer, intestinal ulcers, effects of stroke, urinary incontinence, bowel disease, mood disorder or anxiety. **Mean ADG Score - a weighted score based on an individual’s aggregated diagnosis groups (ADG). Austin’s weighted ADG score has been described and validated elsewhere. Percentages represent “percent responded”. Sampling weights were used to produce population estimates.

**Figure 1:**
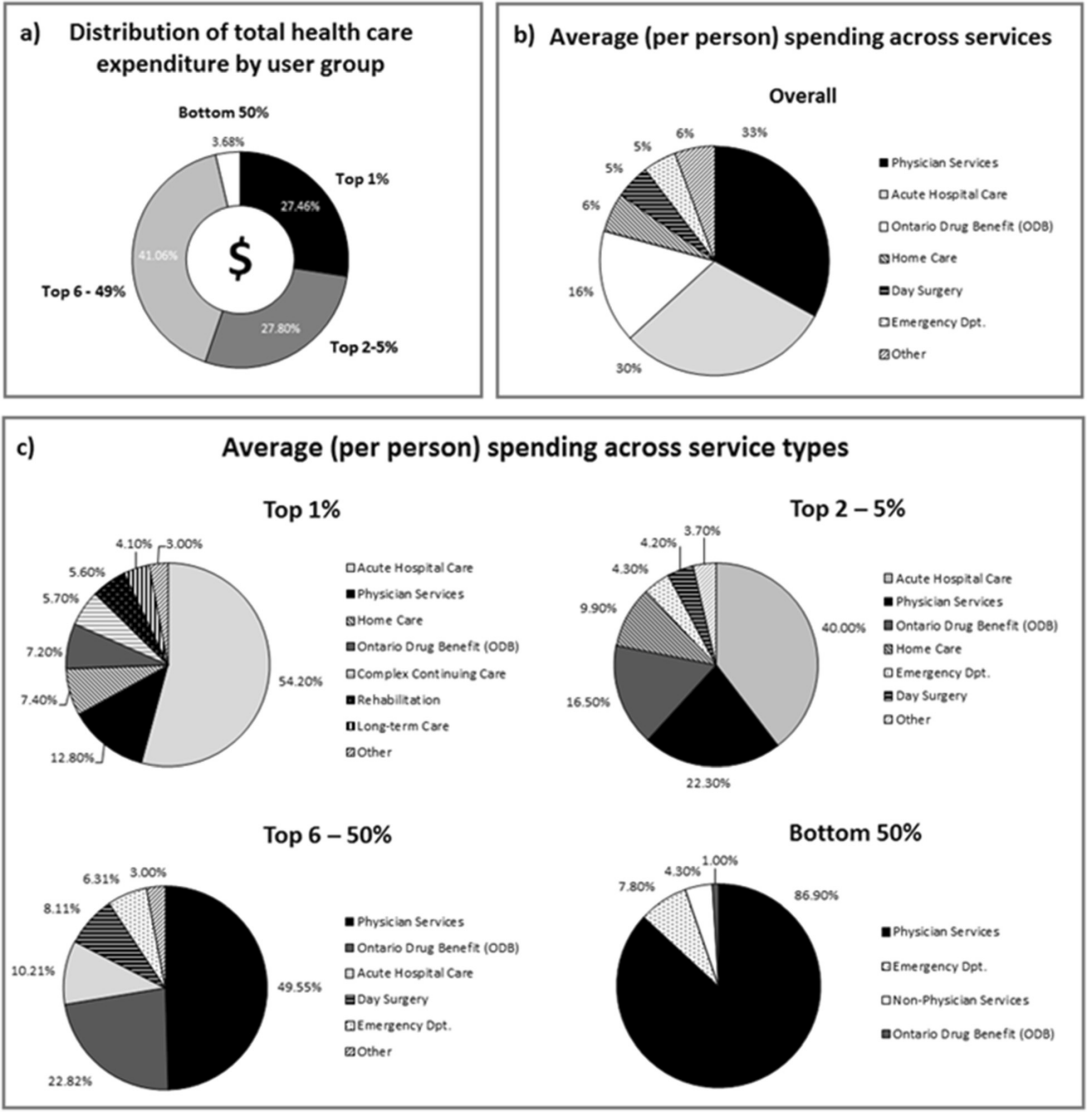
**Distribution of Health Care Spending.** The proportion of total health care spending incurred by each user group **(a)** and average (per person) spending across health care sectors for the overall weighted population **(b)** and by user group **(c)**.

As expected, HCU accounted for the greatest proportion of health care spending within the non-institutionalized community. The Top 1% accounted for 27.5% of total health care spending, which translates to over $15 Billion (95% CI: $13.5 – 16.9 B) in health care costs (Figure 1a). Combined, the top 5% of users incurred over 55% of total health care costs. In comparison, the Bottom 50% accounted for less than 4% of total spending (approximately $2 billion). In the overall sample, 33.0% [95% CI: (31.4%, 32.9%)] of health care costs were incurred through physician services, 30.2% [95% CI: (28.8%, 31.6%)] through acute hospital care and 16.0% (95% CI: 15.4, 16.6) was spent on eligible drug prescriptions (ODB) (Figure 1b). In comparison, the largest contributor for the Top 1% was acute hospital care spending, accounting for an overwhelming 54.2% [95% CI: (51.1%, 57.4%)] of total costs (Figure 1c). For the Top 2-5%, acute hospital care accounted for 40.0% [95% CI: (38.8%, 41.2%) of total spending; while for the Top 6-50% group, acute hospital care accounted for only 10.2% [95% CI: (9.6%, 10.8%)] of the total costs incurred. The Bottom 50% incurred no costs on acute hospital care and instead expenditures mainly occurred through physician services [86.9%; 95% CI: (85.9%, 87.7%)].

For the overall weighted sample, average per-person spending was just under $2,000. Average spending was $142 for the Bottom 50% (Table 2), while the Top 1% incurred on average $53,150; a nearly 400-fold difference. Unsurprisingly, the highest average costs for the HCU groups corresponded to acute hospital care, while physician services were the largest contributor to average expenses for the Top 6-50% and Bottom 50% groups. Interestingly, only 75% of the Bottom 50% saw a physician during the follow-up year (Table 3). All four user groups were similarly likely to be enrolled with a physician not compensated through the typical fee-for service payment system (capitation services are provided through Family Health Networks/Organizations in Ontario).

**Table 2:** Average (per person) expenditure* across health care service types for the weighted CCHS sample

**Service type**	**Overall (95% CI)**	**Top 1% $CAD (95% CI)**	**Top 2 – 5% $CAD (95% CI)**	**Top 6 – 50% $CAD (95% CI)**	**Bottom 50% $CAD (95% CI)**
**Acute hospital care**	$ 584 (542 – 627)	$ 28,830 (25,620 – 32,040)	$ 5,382 (5,185 – 5,578)	$ 180 (167 – 192)	$ 0 --
**Physician services**	$ 639 (627 – 650)	$ 6,374 (5,920 – 6,827)	$ 2,995 (2,904 – 3,088)	$ 874 (863 – 885)	$ 124 (121 – 126)
**Ontario drug Benefit (ODB)** ^**†**^	$ 309 (298 – 319)	$ 3,803 (3,179 – 4,428)	$ 2,214 (2,071 – 2,357)	$ 403 (391 – 415)	$ 1 (1 – 2)
**Home care**	$ 107 (98 – 117)	$ 3,910 (3,261 – 4,560)	$ 1,337 (1,192 – 1,482)	$ 32 (29 – 36)	$ 0 --
**Emergency Dpt.**	$ 91 (89 – 94)	$ 1,271 (1255 – 1,387)	$ 584 (556 – 612)	$ 111 (107 – 114)	$11 (10 – 12)
**Day surgery**	$ 95 (91 – 99)	$ 744 (554 – 934)	$ 570 (523 – 618)	$ 143 (137 – 149)	$ 0 --
**Complex continuing care**	$ 32 (21 – 44)	$ 3,007 (1,848 – 4,167)	$ 54 (30 – 78)	$ 0 --	$ 0 --
**Rehabilitation**	$ 36 (27 – 44)	$ 2,967 (2,156 – 3,779)	$ 150 (107 – 193)	$ 0 --	$ 0 --
**Long-term care**	$27 (21 – 32)	$2,172 (1,627 – 2,717)	$123 (84 – 162)	$0 (0 – 1)	$0 --
**Non-Physician Services** ^**‡**^	$ 15 (15 – 16)	$ 70 (51 – 88)	$ 42 (35 – 48)	$ 22 (21 – 24)	$ 6 (6 – 6)
**All services**	$ 1,935 (1,872 – 1,997)	$ 53,150 (49,518 – 56,783)	$ 13,450 (13,234 – 13,666)	$ 1,765 (1,741 – 1,790)	$ 142 (140 – 144)

*Expenditure ($CAD) calculated for the year following CCHS interview. ^**†**^Individuals eligible for ODB: Ontarians 65 years of age and older, those receiving Ontario Works (a financial assistance program), on the Ontario Disability Support Program, live in LTC, etc. ^**‡**^Non-Physician services include optometrists, physiotherapists, etc. for covered individuals (Ontarians 65 years or age or older, those with specific chronic diseases, and those in specific government assistance programs).

**Table 3:** **Proportion of the weighted CCHS sample, according to cost rank groups, using each health care service type**

	**Proportion of the population or cost-rank group using health care sector (%)**				
**Sector**	**Overall % (95% CI)**	**Top 1% % (95% CI)**	**Top 2 – 5% % (95% CI)**	**Top 6 – 50% % (95% CI)**	**Bottom 50% % (95% CI)**
Total population	N =28,529,265	N =285,255	N =1,141,128	N =12,839,271	N =28,529,265
Physician services	87.4 (87.0, 87.8)	100.0 (100, 100)	100.0 (99.9, 100)	99.7 (99.6, 99.9)	75.0 (74.3, 75.7)
Rehabilitation	24.2 (20.3, 28.1)	13.8 (11.2, 16.3)	2.6 (1.8, 3.3)	0	0
Long-term care	23.2 (18.9, 27.4)	14.1 (11.3, 17.0)	2.1 (1.3, 2.9)	0	0
Emergency Dpt.	21.4 (21.0, 21.8)	83.5 (79.8, 87.2)	66.1 (64.1, 68.2)	31.2 (30.5, 32.0)	7.8 (7.4, 8.1)
Non-Physician services	20.8 (20.4, 21.2)	36.2 (31.8, 40.6)	38.1 (36.1, 40.1)	28.6 (27.9, 29.3)	12.0 (11.5, 12.5)
Ontario Drug Benefit	20.1 (19.8, 20.3)	84.4 (80.6, 88.1)	70.7 (68.7, 72.7)	33.5 (32.9, 34.1)	2.7 (2.4, 2.9)
Complex continuing care	13.9 (11.2, 16.5)	10.1 (8.0, 12.3)	0.9 (0.5, 1.2)	0	0
Physician services - Capitation*	12.7 (12.4, 13.0)	14.4 (11.9, 16.9)	13.8 (12.5, 15.1)	13.6 (13.1, 14.0)	11.7 (11.3, 12.1)
Day surgery	9.7 (9.4, 10.0)	37.0 (32.1, 42.0)	36.8 (34.8, 38.8)	17.4 (16.8, 18.0)	0.1 (0, 0.1)
Acute hospital care	6.5 (6.3, 6.8)	92.0 (89.6, 94.3)	71.7 (69.7, 73.7)	6.1 (5.7, 6.5)	0
Home care	3.3 (3.2, 3.5)	68.0 (63.7, 72.4)	38.3 (36.2, 40.4)	2.5 (2.3, 2.7)	0
Any service	90.1 (89.8, 90.5)	100.0 (100, 100)	100.0 (100, 100)	100.0 (100, 100)	80.3 (79.6, 80.9)

*In contrast to the typical fee-for-service billing system, physicians in the capitation system work in family health teams/organizations and receive a flat rate fee for each patient served regardless of the number or type of services provided.

Table 4 summarizes the results of the unadjusted, age-adjusted and fully-adjusted multinomial regression models. The proportional odds assumption was tested and was found to be significantly violated (p < .0001); thus, confirming the appropriateness of treating the outcome as a categorical, opposed to an ordered, response. Of the socio-demographic variables, age had the strongest association with increasing levels of health care utilization. In the fully adjusted model, the effects of age were largely reduced but still remained significant. Compared to men, women were slightly more likely to be community-dwelling HCU, but were twice as likely to be moderate-cost users (Top 6-50%). While visible minority status was found to be significantly associated with low-cost utilization, no trend across levels of users groups was noted. In the adjusted models, these effects were reduced and in the case of the Top 6-50% group, the association between visible minority status and health care use were reversed. Lower income had a strong relationship with utilization in the unadjusted models. However in the full-adjustment models, extreme HCU was not significantly related to income and other utilization groups showed a non-linear pattern, with middle-income group having lower likelihood and the lowest-income group having higher likelihood of higher utilization (compared to the highest income group).

**Table 4:** **Weighted unadjusted, age-adjusted and adjusted odds ratios (and corresponding 95% confidence intervals) according to multinomial logistic analysis**

**Characteristic**	Top 1%*			**Top 2** – 5%*			**Top 6** – 50%*		
	**Un-adjusted**	**Age-adjusted**	**Adjusted**	**Un-adjusted**	**Age-adjusted**	**Adjusted**	**Un-adjusted**	**Age-adjusted**	**Adjusted**
**Demographics & Socioeconomics**									
**Sex**									
Female vs. Male	1.34 (1.10–1.64)	1.47 (1.19–1.81)	1.27 (1.01–1.59)	1.78 (1.62–1.95)	1.93 (1.74–2.13)	1.71 (1.53–1.92)	2.09 (2.00–2.18)	2.20 (2.09–2.31)	1.97 (1.86–2.08)
**Age group**									
18 – 34	1.00 (Ref)	--	1.00 (Ref)	1.00 (Ref)	--	1.00 (Ref)	1.00 (Ref)	--	1.00 (Ref)
35 – 49	3.14 (1.71–5.77)	--	1.91 (1.01–3.60)	1.62 (1.33–1.98)	--	1.05 (0.84–1.33)	1.37 (1.29–1.45)	--	1.04 (0.97–1.12)
50 – 64	12.22 (6.86–21.76)	--	3.67 (1.93–6.99)	4.60 (3.89–5.44)	--	1.67 (1.37–2.05)	2.50 (2.35–2.66)	--	1.42 (1.31–1.53)
65 – 74	113.39 (66.15–194.37)	--	27.92 (15.00–51.95)	38.92 (32.47–46.65)	--	11.51 (9.25–14.33)	12.77 (11.65–13.99)	--	6.45 (5.81–7.17)
75+	501.66 (283.42–887.95)	--	67.17 (35.53–127.98)	141.84 (115.88–173.60)	--	26.48 (20.71–33.85)	23.57 (20.47–27.15)	--	9.64 (8.30–11.20)
**Ethnicity**									
Visible Minority vs. White	0.44 (0.28–0.71)	0.87 (0.54–1.40)	0.82 (0.49–1.39)	0.44 (0.36-0.54)	0.74 (0.60–0.91)	0.74 (0.57–0.95)	0.89 (0.83–0.95)	1.14 (1.06–1.23)	1.13 (1.02–1.25)
**Immigrant status**									
Immigrant vs. Canadian-Born	1.10 (0.87–1.40)	0.83 (0.65–1.06)	0.83 (0.63–1.09)	1.07 (0.96–1.19)	0.84 (0.75–0.94)	0.92 (0.81–1.04)	1.28 (1.21–1.35)	1.11 (1.05–1.18)	1.10 (1.02–1.18)
**Marital status**									
Other vs. Married^‡^	1.18 (0.97–1.43)	1.42 (1.15–1.75)	1.25 (1.00–1.56)	1.03 (0.94–1.14)	1.23 (1.10–1.37)	1.04 (0.92–1.17)	0.77 (0.74–0.81)	0.92 (0.88–0.97)	0.87 (0.82–0.92)
**Household income**									
High	1.00 (Ref)	1.00 (Ref)	1.00 (Ref)	1.00 (Ref)	1.00 (Ref)	1.00 (Ref)	1.00 (Ref)	1.00 (Ref)	1.00 (Ref)
Middle-High	1.25 (0.77–2.03)	1.23 (0.76–1.99)	0.98 (0.60–1.59)	1.16 (0.95–1.41)	1.14 (0.94–1.09)	0.99 (0.80–1.21)	1.06 (0.98–1.14)	1.07 (0.99–1.16)	1.00 (0.92–1.09)
Middle	2.07 (1.31–3.28)	1.61 (1.02–2.56)	1.06 (0.66–1.72)	1.27 (1.06–1.51)	1.04 (0.87–1.25)	0.79 (0.65–0.97)	1.09 (1.02–1.16)	1.01 (0.94–1.09)	0.88 (0.81–0.95)
Middle-Low	2.95 (1.90–4.58)	1.82 (1.18–2.82)	1.03 (0.66–1.61)	2.12 (1.77–2.53)	1.44 (1.20–1.74)	0.97 (0.80–1.19)	1.36 (1.27–1.46)	1.17 (1.08–1.26)	0.94 (0.86–1.02)
Low	6.68 (4.35-10.24)	3.86 (2.49–5.97)	1.47 (0.94–2.30)	4.13 (3.49–4.90)	2.66 (2.23–3.19)	1.35 (1.11–1.65)	1.90 (1.76–2.05)	1.60 (1.47–1.74)	1.14 (1.04–1.25)
**Health status**									
**Has a regular doctor**									
Yes vs. No	2.63 (1.59–4.38)	1.51 (0.90–2.54)	1.22 (0.68–2.19)	3.23 (2.56–3.85)	2.07 (1.65–2.60)	1.61 (1.25–2.07)	2.59 (2.33–2.78)	2.02 (1.84–2.23)	1.55 (1.40–1.71)
**Has a self-reported chronic disease**									
Yes vs. No	20.02 (14.43–27.77)	8.22 (5.81–11.6)	2.53 (1.75–3.67)	9.68 (8.39–11.17)	4.90 (4.24–5.66)	2.15 (1.85–2.50)	3.43 (3.27–3.59)	2.42 (2.30–2.55)	1.75 (1.66–1.85)
**ADG Score - Quintile**									
Q 1 (Lowest)	1.00 (Ref)	1.00 (Ref)	1.00 (Ref)	1.00 (Ref)	1.00 (Ref)	1.00 (Ref)	1.00 (Ref)	1.00 (Ref)	1.00 (Ref)
Q 2	1.30 (0.59–2.87)	0.93 (0.41–2.09)	1.07 (0.46–2.47)	0.53 (0.41–0.69)	0.42 (0.33–0.55)	0.53 (0.40–0.70)	0.49 (0.45–0.53)	0.43 (0.40–0.47)	0.57 (0.52–0.62)
Q 3	2.04 (1.03–4.03)	1.25 (0.62–2.52)	1.24 (0.60–2.53)	1.26 (0.96–1.66)	0.91 (0.69–1.20)	0.97 (0.73–1.30)	0.97 (0.90–1.05)	0.81 (0.75–0.88)	0.93 (0.85–1.01)
Q 4	4.81 (2.78–8.32)	1.97 (1.11–3.49)	1.59 (0.88–2.85)	2.96 (2.38–3.69)	1.56 (1.24–1.95)	1.43 (1.13–1.81)	1.75 (1.64–1.88)	1.22 (1.13–1.31)	1.27 (1.18–1.38)
Q 5 (Highest)	92.96 (55.55–155.58)	22.37 (12.77–39.16)	10.16 (5.77–17.90)	25.94 (21.19–31.77)	8.68 (6.99–10.78)	5.36 (4.30–6.68)	5.26 (4.90–5.66)	2.79 (2.57–3.02)	2.51 (2.31–2.74)
**Body mass index**									
Underweight	1.97 (1.33–2.91)	2.82 (1.84–4.33)	1.40 (0.88–2.23)	1.70 (1.29–2.24)	2.28 (1.67–3.11)	1.37 (0.97–1.93)	1.06 (0.91–1.25)	1.29 (1.09–1.53)	0.99 (0.82–1.19)
Normal weight	1.00 (Ref)	1.00 (Ref)	1.00 (Ref)	1.00 (Ref)	1.00 (Ref)	1.00 (Ref)	1.00 (Ref)	1.00 (Ref)	1.00 (Ref)
Overweight	1.27 (1.01–1.60)	0.98 (0.77–1.24)	0.93 (0.73–1.19)	1.32 (1.18-1.47)	1.05 (0.93–1.18)	1.02 (0.90–1.15)	1.14 (1.08–1.19)	0.96 (0.91–1.01)	1.01 (0.95–1.07)
Obese	2.00 (1.54–2.59)	1.96 (1.50–2.57)	1.09 (0.82–1.46)	2.13 (1.90–2.39)	2.06 (1.82–2.34)	1.33 (1.15–1.53)	1.53 (1.44–1.64)	1.40 (1.31–1.50)	1.21 (1.13–1.31)
**Self-perceived general health**									
Good	1.00 (Ref)	1.00 (Ref)	1.00 (Ref)	1.00 (Ref)	1.00 (Ref)	1.00 (Ref)	1.00 (Ref)	1.00 (Ref)	1.00 (Ref)
Fair	16.10 (13.14–19.74)	9.62 (7.76–11.9)	4.01 (3.17–5.07)	9.42 (8.30–10.68)	6.14 (5.36–7.04)	2.93 (2.53–3.40)	3.41 (3.12–3.71)	2.68 (2.44–2.94)	1.69 (1.52–1.87)
Poor	173.62 (131.35–229.50)	109.37 (81.57–146.64)	26.44 (18.93–36.94)	67.16 (54.62–82.59)	45.77 (36.62–57.20)	13.64 (10.75–17.31)	10.90 (9.04–13.13)	8.55 (7.04–10.4)	3.99 (3.24–4.91)
**Self-perceived mental health**									
Not Good vs. Good	6.21 (4.55–8.48)	8.32 (6.03–11.5)	1.32 (0.91–1.90)	4.20 (3.57–4.94)	5.31 (4.46–6.32)	1.22 (1.00–1.49)	2.27 (2.03–2.55)	2.52 (2.23–2.83)	1.23 (1.08–1.40)
**Consulted mental health professional**									
Yes vs. No	3.19 (2.37–4.29)	6.91 (5.07–9.42)	2.43 (1.72–3.44)	2.67 (2.33–3.05)	4.81 (4.15–5.58)	2.00 (1.69–2.37)	2.14 (1.98–2.32)	2.74 (2.52–2.97)	1.67 (1.52–1.83)
**Health behavior**									
**Life stress**									
High vs. Low	1.18 (0.93–1.51)	2.47 (1.90–3.20)	1.01 (0.78–1.29)	1.09 (0.98–1.21)	1.92 (1.71–2.16)	1.08 (0.95–1.23)	1.04 (0.98–1.10)	1.31 (1.24–1.39)	1.06 (0.99–1.13)
**Smoking status**									
Non-smoker	1.00 (Ref)	1.00 (Ref)	1.00 (Ref)	1.00 (Ref)	1.00 (Ref)	1.00 (Ref)	1.00 (Ref)	1.00 (Ref)	1.00 (Ref)
Former (Daily)	2.74 (2.18–3.44)	1.57 (1.24–2.00)	1.35 (1.07–1.70)	2.38 (2.14–2.65)	1.48 (1.32–1.66)	1.38 (1.23–1.55)	1.52 (1.43–1.60)	1.12 (1.05–1.19)	1.15 (1.08–1.22)
Light smoker	0.66 (0.50–0.87)	1.28 (0.95–1.73)	0.91 (0.66–1.25)	0.76 (0.66–0.87)	1.24 (1.07–1.44)	1.03 (0.88–1.21)	0.79 (0.74–0.85)	0.97 (0.91–1.04)	0.99 (0.91–1.07)
Heavy smoker	1.67 (1.15–2.43)	2.66 (1.80–3.95)	1.19 (0.77–1.86)	1.39 (1.18–1.65)	1.95 (1.63–2.34)	1.19 (0.97–1.46)	0.83 (0.75–0.90)	0.89 (0.81–0.98)	0.87 (0.78–0.96)
**Physical activity**									
Active vs. inactive	0.39 (0.32–0.48)	0.46 (0.37–0.57)	0.74 (0.59–0.93)	0.57 (0.52–0.64)	0.65 (0.58–0.72)	0.92 (0.82–1.04)	0.78 (0.75–0.82)	0.83 (0.79–0.87)	0.95 (0.90–1.01)
**Alcohol consumption (past 12 months)**									
Non-Drinker vs. Current drinker	4.02 (3.26–4.96)	2.80 (2.24–3.50)	1.57 (1.26–1.97)	2.97 (2.68–3.29)	2.21 (1.98–2.47)	1.46 (1.28–1.66)	1.68 (1.58–1.79)	1.46 (1.37–1.56)	1.12 (1.04–1.21)

*Referent group: Bottom 50% of users. ^‡^Other: divorced, separated, widowed or single (never married); Married includes common-law.

Variables capturing health status (both self-reported and measured by health care utilization) showed very strong associations with health care consumption. Having a self-reported chronic disease, ADG score in the highest quintile, and reporting poorer self-perceived general health were strongly associated with increased health care utilization. The adjusted odds ratios for these variables were greatly reduced, but still remained largely influential. Similarly, recent contact with a mental health professional and poorer self-perceived mental health was also strongly associated with increased healthcare spending; however, these effects were strengthened in the age-adjusted model. Having a regular medical doctor showed protective effects; these remained mostly significant, even with attenuation after adjustment. In the unadjusted analysis, all unhealthy weight categories were associated with increased health care costs; the odds of being a community-dwelling HCU were two times greater for underweight or obese individuals as compared to normal weight individuals. Further, HCU was associated with former smoking status, being physically inactive and being a current non-drinker; life stress, however, was not significantly associated with use. Overall, the health behavior variables were only moderately associated with HCU and effect sizes were greatly reduced after adjustment.

In the sensitivity analysis, the inclusion of additional measures of SES, including individual- and ecological-level variables, had no effect on any of the multinomial model estimates. This suggests that the multiple individual-level SES indicators originally included in the fully-adjusted multinomial regression model are quite robust towards characterizing HCU in this non-institutionalized, community-dwelling population.

## Discussion

This study offers a full characterization of the broad range of social, behavioral, and health factors associated with health care utilization for a population-based sample in the context of a universal health care system. While there has been a recent renewed interest in health care spending, sustainability, quality of care and patient outcomes, focus on the broader characterization of these individuals that would support alternative perspectives on how to best address this issue has been limited. For example, HCU interventions have been overwhelmingly focused on case-management for patients who are already HCU or frequent users of the health care system; knowledge of the upstream determinants of HCU, particularly those that are non-clinical in nature, such as SES and health behaviors, is desperately lacking. Recent reports have suggested that health programs targeting high-risk groups may play an important role in health care sustainability [17-19], suggesting that $1.5 billion could be saved if just a 10% reduction in spending could be achieved for Ontario’s Top 5% of spenders [1]. Understanding HCU from a broader socio-economic and cultural perspective is crucial if modifiable characteristics are to be identified and addressed from both within and outside the health care system.

The results of this study are consistent with previous research on HCU. Many studies have attempted to characterize HCU; however, these have examined only a minimal number and breadth of variables, have relied on binary definitions of HCU and have employed simplistic analytic techniques. Indeed, most characterizations of the HCU population have been limited to the information available in administrative data sources, such as age, sex, ethnicity and clinical measures; while others have included SES, most examined only income or education and relied on ecological-level measures. Only one other study has investigated HCU in Canada using health survey data linked to medical utilization records [2]. According to this 2009 study by Lemstra et al., low-income residents of Saskatoon had higher health care costs overall compared to higher-income groups. This is in agreement with the findings of our study; however, our affirmation of this association was confirmed even after controlling for several additional variables and finer categories of utilization. These findings suggest that differences in health care spending are not merely a result of differences in health-seeking behavior, but may reflect higher needs in specific groups, such as low income users, as a result of poorer health. Our results are also consistent with previous literature examining socioeconomic status (SES) and HCU [2-16], although none of the previous studies adjusted for the number and depth of individual-level SES variables that were included in our study [2-16]. In the descriptive and unadjusted multinomial analyses, our study found gradients existed across multiple dimensions of SES and were strongly and significantly associated with increased health care costs. However, adjusting for confounders resulted in mostly non-significant associations and the attenuation of the incremental relationship seen with household income. This demonstrates the importance of controlling for confounders when interpreting associations with HCU – as opposed to simply looking at descriptive characteristics, as often done in HCU studies. Unlike previous studies, which have typically described health care utilization as a binary outcome (i.e. HCU vs. non-HCU), we were able to further dissect gradients of use by applying a multinomial model. These results suggest that the HCU population is not homogenous and the finer separation is important to further understanding the associations. For instance, the associations among the extreme HCU (top 1%) are significantly stronger than, and in some cases differ from, that of the Top 2-5% HCU.

This current study is particularly novel in that we have investigated the effects of heath behaviors and health status in addition to multiple socio-demographics measures. We confirmed health status, primarily ADG score, and increased age to be strongly associated with increasing levels of health care utilization. What is unique compared to other studies is that we were able to demonstrate the significance of this association across self-reported indicators of health status, in addition to those identified through medical claims. Findings related to self-reported health (physical and mental) provide an interesting perspective on the self-perceptions of those individuals who are in the highest HCU group compared to those in the lowest. The strong associations seen, even after adjustment for multiple co-morbidities, demonstrate that every incremental utilization group above the bottom 50th percentile were more likely to classify their health as poor. These findings were particularly evident for self-reported general health, which demonstrated a stronger magnitude of effect than even the clinically-derived ADG score. The research on the patient perspective of HCU is very limited and would be an important area of further study and may have useful implications for HCU intervention and policy.

This study uniquely enhances our understanding of HCU through the investigation of health behaviors. Behavioral factors, such as physical activity, may be more amenable to change than others, particularly SES, and thus, more likely to have implications for interventions or policies targeted at enabling healthy choices. We did not find that established risky health behaviors, such as smoking and alcohol consumption [25], to be overwhelming drivers of short term HCU gradients. This finding, however, is likely an artifact of the short-term follow-up period. It is reasonable that as an individual’s health declines, medical contraindications and healthy living recommendations would affect health behavior choices. For example, HCU may be more likely to quit smoking or drinking alcohol upon recommendation by their doctor or due to contraindications of ongoing treatment. This could explain why HCU was associated with former smoker and current non-drinker status in our study. A longer follow-up examining trajectories of health care utilization is necessary to further study the health effects of such behaviors.

### Limitations

This study is strengthened by the novel use of a large, linked population survey sample to more broadly characterize the non-institutionalized, community-dwelling HCU population and the use of a multinomial analysis to further dissect trends across HCU groups. However, there are some limitations that must be mentioned. Particularly, the CCHS sampling frame excludes the institutionalized, persons living on Aboriginal reserves, full-time members of the Canadian Forces and persons living in certain remote areas (approximately 2% of the Canadian population) [20]. As a result, Ontarians not living in private dwellings, individuals residing in LTC or complex continuing care facilities, mental health institutions or hospitals at the time of interview are excluded from these analyses. It is expected that a number of Ontario’s HCU reside in these facilities, and would not be represented by the CCHS. Indeed, long-term care (LTC) spending accounted for less than 5% of HCU spending in our analysis, and provincial estimates suggest this proportion to be much higher [1,26]. This would affect external generalizability to the broader Ontario population, but not the internal validity since we ranked within the CCHS population and not within the entire population so relative cost categories are accurate within the study population. Similarly, homeless Ontarians would have been excluded from the CCHS. Given the relationship between SES and HCU, it is likely that a portion of HCU in Ontario are homeless and are not represented in the current study. While individuals residing within these institutions or who were homeless at baseline would not be captured by the CCHS, all CCHS respondents who transferred into these facilities or became homeless following CCHS interview would be captured, and thus, their health care costs also captured in this study [21].

Further, the CCHS-RPDB linkage is conducted only for those respondents who agreed to linkage and provided a valid health care number; selective agreement and low coverage rates may lead to biased linked samples. An evaluation of the CCHS Cycle 1.1 linkage observed that nearly 91% of Ontario CCHS respondents agreed to linkage, but only 70% agreed to linkage and provided a valid health card number; however, among Ontarian respondents who were hospitalized, over 91% of respondents agreed to linkage and provided a valid health card number [27]. Therefore, use of the Ontario linked CCHS cohort for health services research shows acceptable coverage, but this potential for bias should be considered when interpreting estimates from the CCHS linked file. Also, because the CCHS relies on self-reported data there is potential for reporting bias, such as recall or social desirability bias. Additionally, because not every question is asked in each CCHS cycle and certain questions are only asked in select provinces, we were limited in which variables to include. For instance, the Health Utilities Index (HUI®), a health status variable incorporating both qualitative and quantitative features to provide a summary measure of individual health, was not available for all three cycles and therefore could not be included, despite its relevance [28].

Furthermore, the time frame of the study is such that we characterize patients as HCU in the year following interview, although the nature of them being high-users may have influenced some of the self-reported information, i.e. reverse causality [29]. This may explain why a weak association between HCU and health behavior was noted in this study. However, the purpose of this study is to more fully characterize this population, and not to infer causality, so this is only a minor limitation to our study design.

Lastly, the health care expenditures included in this analysis are limited to only those covered by Ontario’s universal health insurance plan, OHIP. Except for eligible members of the adult population (e.g. those over 65, receiving government assistance or with specific diseases) OHIP coverage excludes prescription drug costs (outside of those received in hospital), allied health services (physiotherapy, registered massage therapy, etc.), dental care, eye care, and assistive devices (e.g. crutches, splints, and casts). However, compared to costs associated with acute hospital care and physician services, these represent a relatively smaller proportion of health care spending.

## Conclusion

This study has corroborated the findings of previous research, and has provided new information towards understanding a broad range of characteristics associated with increased health care costs that have not been well characterized in the literature. We found that community-dwelling HCU tended to be older with multiple comorbidities and were also more likely to be white, female, and have lower household income. We also showed the importance of self-rated health, both mental and general, and the presence of self-reported chronic conditions. The findings of this study may help guide future work to identify populations at risk of becoming HCU and provides information that would allow for policies or interventions to be better informed by the economic, social, health status and behavioral profile of community-dwelling HCU. Further research looking at the trajectory of HCU over time will allow for a better understanding of the upstream determinants of health care utilization. This and future research will aid in identifying modifiable and addressable factors associated with becoming a HCU, and thus will assist efforts to identify populations at-risk of becoming HCU. Understanding the broader determinants of HCU is crucial to informing policy decisions addressing the common medical and public health goal of improving population health, and reaching the health care targets of sustainability, better quality of care, and improved patient outcomes.

## Abbreviations

HCU: High-cost user(s)
CCHS: Canadian Community Health Survey
ADG: Aggregated Diagnosis Groups
